# Prevalence of Overweight and Obesity in the Era of CFTR Modulators in Patients with Cystic Fibrosis

**DOI:** 10.3390/nu18050755

**Published:** 2026-02-26

**Authors:** Anam Bashir, Mary Bridget Kastl, Xingmei Wang, Laura Padula, Elizabeth Reid, Rachel Kofsky, Nikhil Pai, Maria Mascarenhas

**Affiliations:** 1 Division of Gastroenterology, Hepatology & Nutrition, Children’s Hospital of Philadelphia, Philadelphia, PA 19104, USA; 2 Division of Pulmonology, Children’s Hospital of Philadelphia, Philadelphia, PA 19104, USA; 3 Center for Clinical Epidemiology and Biostatistics, Perelman School of Medicine, University of Pennsylvania, Philadelphia, PA 19104, USA; 4 Division of Clinical Nutrition, Children’s Hospital of Philadelphia, Philadelphia, PA 19104, USA; 5 Department of Pediatrics, Perelman School of Medicine, University of Pennsylvania, Philadelphia, PA 19104, USA; 6 Center for Microbial Medicine, Children’s Hospital of Philadelphia, Philadelphia, PA 19104, USA

**Keywords:** obesity, cystic fibrosis, metabolic complications, CFTR modulators, pancreatic insufficiency, fat malabsorption

## Abstract

**Background/Objectives**: Cystic fibrosis (CF) is a genetic disorder historically associated with malnutrition. The advent of CF transmembrane conductance regulator (CFTR) modulators and improved pulmonary outcomes have coincided with increased body mass index (BMI). This study aims to evaluate the prevalence of overweight/obesity among children with cystic fibrosis and associated comorbidities. **Methods**: A retrospective chart review assessed patients with CF (2–23 years of age). Data collected included demographics, CF genotype, anthropometric measurements, pancreatic function, medication history, and any comorbid diagnoses. BMI categories were defined as: underweight (<5th percentile), normal weight (5th–85th percentile), overweight (85th–95th percentile), and obese (>95th percentile). **Results**: Among 243 patients (mean age 10.4 years, 53% male), 4 (1.6%) were malnourished, 192 (79%) had normal weight, and 47 (19.3%) were overweight (OW)/obese (26 (10.7%) OW, and 21 (8.6%) were obese). OW/obese patients (mean age 11.3 years, 61.7% male) included 20 patients (42.5%) with two severe CF-related mutations, 21 (44%) with pancreatic insufficiency, and 40 (85.1%) receiving CFTR modulator treatment. Obstructive sleep apnea (OSA) was the most common comorbidity in OW/obese patients with CF, followed by impaired glucose intolerance and CF-related diabetes and hyperlipidemia; these associations did not reach statistical significance. **Conclusions**: Overweight/obesity affects nearly one in five patients with CF, including those with severe genotype and pancreatic insufficiency.

## 1. Introduction

Cystic fibrosis (CF) is an autosomal recessive, genetic, multisystem disorder caused by mutations in the cystic fibrosis transmembrane conductance regulator (CFTR) gene [1]. It is one of the most common genetic diseases in the Caucasian population, affecting 1 in 10,000 individuals throughout the world [2]. The CFTR gene encodes for the CFTR protein, which is a chloride and bicarbonate channel in various organs like sweat glands, respiratory tract, gastrointestinal tract, and reproductive tracts. CFTR dysfunction leads to pancreatic insufficiency, diffuse bronchiectasis with chronic respiratory failure, liver disease, pulmonary infections, and infertility. Historically, CF has been associated with a range of nutritional challenges, most notably malnutrition, due to pancreatic insufficiency, altered gastrointestinal function, and increased energy expenditure from chronic lung disease. Undernutrition in CF is known to negatively impact lung function, worsen clinical outcomes, and increase mortality rates [3]. Consequently, weight gain is generally considered a positive outcome [4]. Data from CF patient registry indicates that a better forced expiratory volume in one second (FEV1), specifically values of more than 80% predicted or more, is associated with body mass index (BMI) of 50th percentile or more to optimize health outcomes. The CF Foundation thus recommends that children with CF maintain a BMI at or above the 50th percentile [5].

Recent advances in management, including improved respiratory treatments, nutritional interventions, pancreatic enzyme replacement therapy, and the introduction of CFTR modulators have revolutionized the landscape of nutrition care in CF [3]. Based on 2024 CF registry data, life expectancy is estimated to be 65 years [6]. CFTR modulators are used to treat around 80% of people with CF. CFTR modulators restore ion transport, leading to marked improvement in clinical manifestations and lung function. Rates of overweight and obesity in children were increasing prior to CFTR modulators, but, notably, enhanced nutritional status, including increased weight gain, has become even more common with the widespread use of CFTR modulators. However, this has also led to a concerning trend: an increased prevalence of overweight and obesity in individuals with CF, defined as a BMI-for-age of 85th–95th percentile or >95th percentile, respectively. The mechanism of weight gain, particularly with newer CFTR modulator therapies like Elexacaftor/Tezacaftor/Ivacaftor, is likely multifactorial and remains incompletely understood. Decreased energy expenditure, reduced gut inflammation, and improved fat absorption all may play a role [7]. Historically, patients with CF had a shortened life span. However, CFTR modulator therapy has significantly increased life expectancy in this population. As individuals with CF live longer, comorbidities are becoming increasingly relevant [8].

This study aims to estimate the prevalence of obesity and weight-related issues in individuals with CF and assess the burden of comorbidities in this population.

## 2. Materials and Methods

### 2.1. Study Sample

We conducted a retrospective study of patients diagnosed with CF who are currently being treated at Children’s Hospital of Philadelphia (CHOP). The study was approved by the Institutional Review Board (IRB 24-022467). The study was conducted in accordance with the STROBE guidelines for reporting observational research. Patients aged 2–23 years were included in the study.

### 2.2. Study Design

Data collected from patient records included demographic data, CF genotypes (categorized as homozygous F508del, heterozygous F508del, or other), anthropometrics (weight, height, and body mass index), most recent lung function measures (best recent predicted FEV1), medication history, pancreatic status, and comorbid diagnoses. Pancreatic insufficiency was defined by fecal elastase level of <200 ug/g. Patients were classified as underweight if BMI-for-age was <5th percentile, normal weight if BMI was between 5th and 85th percentile, overweight if BMI was between 85th and 95th percentile, and obese if BMI was >95th percentile, as defined by Centers for Disease and Prevention’s sex-specific BMI-for-age growth charts [9]. BMI values were extracted from clinical visits, with the most recent measurement used for analysis. Pulmonary function was evaluated with spirometry, which is routinely performed at our center starting at 4 years of age, and glucose tolerance was evaluated using glucose tolerance testing, also routinely conducted at our center starting at 10 years of age. Impaired glucose tolerance was defined as having a 2 h glucose of 140–199 mg/dL, while CF-related diabetes (CFRD) was defined as having a 2 h glucose of ≥200 mg/dL on a glucose tolerance test based on American Diabetes Association position statement [10]. Obstructive sleep apnea (OSA) was diagnosed through polysomnography. Hypertension was defined as blood pressure ≥ 95th percentile on two separate occasions, adjusted for age, sex, and height percentile.

We assessed age, sex, lung function, pancreatic insufficiency, CFTR modulator use, and F508del genotype (homozygous or heterozygous). Obesity comorbidities were evaluated, including OSA, CFRD, hypertension, and hyperlipidemia. All variables were compared against overweight and obesity status.

All anthropometric variables were measured using standard, calibrated equipment and standardized institutional procedures.

### 2.3. Statistical Analysis

Descriptive statistics summarized demographic and clinical characteristics, with continuous variables reported as means and standard deviations and categorical variables as counts and percentages. Group comparisons between overweight/obese and underweight/normal weight participants used independent *t*-tests for continuous variables and Chi-square or Fisher’s exact tests for categorical variables. Associations between obesity and major cardiometabolic comorbidities were evaluated using contingency table analysis. Pearson’s Chi-square test was applied when expected cell counts met assumptions; for analyses of hypertension, OSA, and hyperlipidemia, Fisher’s exact test was used to account for unavailable data. All tests were two-sided with a significance threshold of *p* < 0.05. All analyses were conducted using SAS 9.4.

## 3. Results

### 3.1. Patient Demographics

A total of 243 patients with CF were included in the study (n = 129 male, 53%; n = 114 female, 46.9%) (Table 1). Mean age was 10.4 years.

Patients were stratified by BMI. Of the 243 patients, 4 (1.6%) were underweight, 192 patients (79%) had normal weight, 47 patients (19.3%) were overweight (OW)/obese, 26 (10.7%) were overweight, and 21 (8.6%) were obese).

### 3.2. Description of Overweight/Obese Patients

Overweight and obesity were prevalent across all age groups, with 23 OW/obese children (49%) under 10 years of age affected, showing a slight male predominance (*p* = 0.0550) (Table 2). Overweight and obesity were observed even in patients with severe mutations: 20 OW/obese patients (42.5%) were homozygous for F508del, and 17 OW/obese patients (36.1%) were heterozygous for F508del (Table 2). Additionally, 21 (44%) of OW/obese patients had pancreatic insufficiency. Among OW/obese patients, 40 (85.1%) were on CFTR modulator therapy (Table 2). Among OW/obese patients on CFTR modulators, 19 (47.5%) patients were already OW/obese prior to starting CFTR modulators, while 21 (52.5%) developed OW/obesity following modulator initiation after a mean duration of 17 months. Pancreatic sufficiency was significantly associated with a higher likelihood of OW/obesity (*p* = 0.0015). Age, sex, lung function, F508del genotype (homozygosity/heterozygosity), and modulator use were not significantly associated with presence of OW/obesity (Table 2).

### 3.3. Prevalence of Overweight/Obesity in Children with Cystic Fibrosis

**Figure 1:**
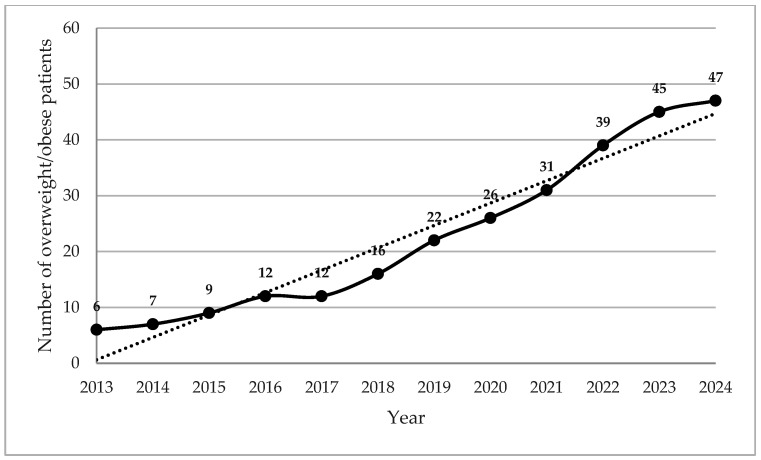
Annual prevalence of overweight/obesity in patients with cystic fibrosis at our institution (2013–2024).

There was a marked upward trend in the prevalence of overweight/obesity over the study period, the number of OW children doubling between 2017 and 2020, tripling by 2022, and quadrupling by 2024. This has continued to trend upwards since this time (Figure 1).

### 3.4. Comorbidities in Overweight/Obese Children with Cystic Fibrosis

Several cardiometabolic comorbidities were associated with OW/obesity in our cohort. Abnormal or impaired glucose tolerance testing (18/28 children evaluated, 64.3%) was most common, followed by OSA (6/47, 12.7%), type 2 diabetes mellitus (4/28, 14%), and hyperlipidemia (1 evaluation, 2.1%). No significant associations were observed between OW/obesity and any cardiometabolic outcomes (*p* > 0.59).

## 4. Discussion

Our study highlights an important shift in the nutritional profile in patients with CF treated at our center, with a considerable portion (19%) classified as overweight or obese. Notably, 8.6% of patients were obese, and 10.7% were overweight, which is comparable to general population, suggesting that children with CF are not protected from the obesity epidemic [11]. Our findings align with previous reports suggesting a rising prevalence of OW and obesity in children with CF. A 2005 UK study reported 7.9% and 1.4% of children homozygous for F508del mutation as overweight and obese, respectively [12]. Similarly, 11.2% of children with CF had a BMI > 85th percentile in Australia (2017), while a Spanish cohort reported 6% overweight and 1% obesity in children with CF in 2017 [13,14]. A US-based center analysis showed 15% of children with CF were overweight and 8% were obese in 2012. In this study, 50% of overweight and 20% of obese patients with CF were pancreatic insufficient [15]. Another study results at a single center that enrolled children with cystic fibrosis 1–19 years of age showed 47% of pancreatic sufficient patients were obese as compared to 22% patients with pancreatic insufficiency [16]. Most recently, the 2023 CF patient registry reported an increase in obesity to 21% in children with CF [6]. Similarly, in adult populations, the prevalence of overweight/obesity has also been noted to increase. A multicenter study in Spain from 2020 to 2023 showed an increase in proportion of overweight and obesity from 8.3% to 22.9%. Significant weight gain was noted in the 12 months after starting CFTR modulator (elexacaftor/tezacaftor/ivacaftor). Lower baseline BMI, lower baseline FEV1 and FVC (forced vital capacity), and higher RV/TLC (residual volume/total lung capacity) value and higher total exacerbations were associated with higher BMI changes [3].

Studies have shown a threshold BMI of up to 23–25 kg/m^2^ for a positive association between BMI and FEV1 in patients with CF [12,17]. Higher adiposity in adults with CF is associated with lower lung function [18]. Higher BMI has been associated with increased insulin resistance in adults with CF. As the life expectancy of this population continues to improve, this relationship may have important implications for their long-term metabolic health as they age [17].

Overweight/obesity was prevalent across all age groups in our cohort with nearly half of OW/obese children being younger than 10 years of age. The early onset of excessive weight gain may suggest that the trend towards excess weight in CF begins in childhood and may persist or potentially worsen into adolescence. Early onset of overweight/obesity with CF raises concerns about the long-term metabolic and pulmonary implications as these patients age, especially as survival rates continue to improve.

Interestingly, overweight/obesity was observed even with more severe CF genotypes. A total of 37 OW/obese patients (78%, *p* = 0.2341) carried at least one F508del, a CF-causing variant that is traditionally associated with more significant disease burden, severe pulmonary disease, pancreatic insufficiency, and greater nutritional challenges. Pancreatic insufficiency, commonly associated with malabsorption and undernutrition, was present in over half of OW/obese patients. The majority (85.1%) of OW/obese patients were on CFTR modulator therapy. Notably, more than half of the OW/obese children became overweight/obese following modulator treatment, while the rest were already overweight/obese before being started on CFTR modulators. These highly effective therapies improve CFTR protein function, leading to better pulmonary outcomes and potentially improved nutritional status. Among the risk factors, pancreatic sufficiency was significantly associated with the risk of overweight/obesity, which may reflect differences in nutrient absorption. However, notably, over half of overweight/obese individuals were pancreatic insufficient, suggesting that other factors like dietary intake, physical activity, and genetic modifiers may have a role to play.

We also observed a rising trend with the number of OW children doubling between 2017 and 2020 and increasing thereon. This rapid rise parallels the increasing use of CFTR modulators and improvements in clinical care.

The potential health implications of overweight and obesity in children with CF are multifaceted. While improved nutritional status is associated with better pulmonary outcomes, excessive weight gain may introduce new risks. Previous research has linked overweight and obesity to OSA in adults with CF, although this association was not found in children [19]. In our cohort, 12.7% of overweight/obese children were diagnosed with OSA, although this association did not meet statistical significance (*p* = 0.53). A study involving 209 adults with CF demonstrated that high BMI is associated with insulin resistance, indicating that overweight status may increase the risk of CFRD [20]. Our cohort revealed a trend towards prevalence of CFRD (14%) and impaired glucose tolerance (25%) among OW and obese patients who had oral glucose tolerance testing performed (*p* > 0.05), which may portend increased metabolic risks as this cohort ages. A study by Harindhanavudhi et al. found that the prevalence of hypertension is higher in overweight/obese patients with CF [21]. Total cholesterol and LDL levels were higher in overweight/obese patients versus normal/underweight patients but remained within normal range [21]. Only one child in our OW/obese population was found to have laboratory evidence of hyperlipidemia. However, lipid panels are not routinely performed in our clinical setting, and thus the true prevalence of dyslipidemia may be underestimated. Our study did not find any children with hypertension.

Several factors may account for the lack of significant associations between OW/obesity and cardiometabolic comorbidities in this pediatric CF cohort. First, cardiometabolic complications of obesity accrue over time, and many participants are likely to have experienced obesity only for a relatively brief period; pediatric data suggest that the cumulative duration of excess adiposity, rather than BMI category alone, is a key determinant of hypertension, dyslipidemia, and glucose dysregulation [22]. Second, our data may suggest the possibility that BMI-defined OW/obesity in CF may not fully reflect underlying cardiometabolic risk. The unique metabolic and inflammatory physiology of cystic fibrosis, together with historically increased energy expenditure and malabsorption, may modify or delay the development of classical obesity-related comorbidities, such that overt hypertension, dyslipidemia, or glucose abnormalities remain uncommon in childhood even among those with elevated BMI [17]. Further studies in larger cohorts, along with additional measures of body composition that better differentiate percent fat mass and lean body mass [23], will be required to further understand the similar prevalence of cardiometabolic comorbidities observed across weight groups in our cohort.

Our findings suggest the need for more individualized and nuanced nutritional counseling that balances adequate caloric intake to support growth and pulmonary health while preventing excessive weight gain. As the survival rates and life expectancy continues to improve, the emerging trend of overweight/obesity may have far reaching implications in this population. Tailored dietary strategies should consider each patient’s genotype, pancreatic function, treatment regimen, metabolic status, and lifestyle factors. This shift will require multidisciplinary collaboration involving dietitians, endocrinologists, pulmonologists, and other specialists to optimize nutritional management and improve long-term health outcomes in this evolving population.

Our study has limitations, including its retrospective design, single-center setting, and lack of detailed data on dietary intake, physical activity, and socioeconomic factors, which may influence nutritional status and comorbidities. We did not evaluate the quality and quantity of patients’ dietary intake. Given that diet can significantly influence metabolic outcomes, its omissions may have influenced the results. The interaction between dietary intake, patient genotype, and treatment modalities were not evaluated, which may have impacted the observed outcomes. We did not perform a comprehensive assessment of all obesity-related complications in our population, potentially missing the presence of certain comorbidities [24]. The relatively short follow-up period after CFTR modulator initiation limits conclusions about the long-term metabolic impact. We did not collect comorbidity data on non-overweight/obese populations and thus cannot conclude whether association of comorbidities is driven by CF and/or overweight/obesity. We acknowledge the absence of multivariate analyses adjusting for potential confounders, such as pancreatic sufficiency and others, primarily due to limitations in sample size and data completeness. Lipid panels are not routinely collected at our center in children with CF and hyperlipidemia may have been underestimated in this population. Due to the limited sample size, subgroup analysis would have lacked power and reliability, and this is an area of future study. Prospective, longitudinal studies with larger cohorts are needed to clarify the mechanisms underlying weight gain, characterize the spectrum of comorbidities among children with CF, assess the consequences of early-onset obesity in this population, and inform the development of targeted interventions.

## 5. Conclusions

One in five patients with CF are overweight/obese. Our findings highlight a critical and emerging issue in CF care—the increasing prevalence of overweight and obesity among children and adolescents. Overweight/obesity is prevalent in children with CF with severe genotypes and pancreatic insufficiency, although no definitive associations could be drawn. The interplay of CFTR modulator therapy, improved nutrition, and other factors has transformed the nutritional landscape, necessitating new clinical approaches to optimize health outcomes in this evolving population. Given the rising prevalence of overweight and obesity among children with CF, traditional nutritional approaches—often centered around high-calorie, high-fat diets designed to combat malnutrition—may no longer be appropriate for all patients.

## Figures and Tables

**Table 1:** Patient demographics.

N	243
Mean Age (years) (SD)	10.4 (5.165)
Sex	
Male	129 (53%)
Female	114 (47%)
Pancreatic Insufficiency	179 (73.6%)
Mutation	
Homozygous F508del	79 (32.5%)
Heterozygous F508del	106 (43.6%)
Other	58 (23.8%)
On Modulator	204 (83.9%)
Type of Modulator	
Elexacaftor/Ivacaftor/Tezacaftor	166 (68.3%)
Ivacaftor	35 (14.4%)
Lumacaftor/Ivacaftor	2 (0.8%)
Tezacaftor/Ivacaftor	1 (0.4%)

**Table 2:** Demographic, genotypic, and clinical characteristics by weight classification.

	Overweight/Obese	Underweight/Normal Weight	*p* Value
N	47 (19.3%)	196 (80.7%)	
Mean Age (years)	11.3	10.5	0.3785
<5 years	5/47 (10.6%)	26/196 (13.2%)	
5–10 years	18/47 (38.2%)	56/196 (28.5%)	
11–16 years	18/47 (38.2%)	87/196 (44.3)	
17–23 years	6/47 (12.7%)	27/196 (13.7%)	
Males	29/47 (61.7%)	100/196 (51%)	0.0550
Females	18/47 (38.2%)	96/196 (48.9%)	
Homozygous F508del (n%)	20/47 (42.5%)	59/196 (30.1%)	0.2341
Heterozygous F508del (n, %)	17/47 (36.1%)	89/196 (45.4%)	
Other	10/47(21.2%)	48/196 (24.4%)	
Pancreatic Sufficiency	21/47 (44%)	43/196 (21%)	0.0015
Mean Recent Best FEV1% (%)	106	102	0.2171
Modulator Use	40/47 (85.1%)	164/196 (83.6%)	0.2770
Elexacaftor/Ivacaftor/Tezacaftor	32/47 (68%)	134/196 (68.3%)	
Ivacaftor	6/47 (12.7%)	29/196 (14.7%)	
Tezacaftor/Ivacaftor	1/47 (2.1%)	-	
Lumacaftor/Ivacaftor	1/47 (2.1%)	1/196 (0.5%)	

## Data Availability

The data presented in this study are available on request from the corresponding author due to privacy or ethical restrictions.

## Abbreviations

The following abbreviations are used in this manuscript:

CF	cystic fibrosis
CFTR Modulator	cystic fibrosis transmembrane conductance regulator modulator
OW	overweight
